# Supplementary material for: Complexome profiling of the Chlamydomonas psb28 mutant reveals TEF5 as an early PSII assembly factor
Source: Plant Cell. 2025 Mar 17;37(6):koaf055. doi: 10.1093/plcell/koaf055 (PMC12164590; doi:10.1093/plcell/koaf055)
Supplement: koaf055_Supplementary_Data [file koaf055_supplementary_data.zip › psb28_interactive.zip!/plots/intensity/qualityPlots/peptides/ASA7_Cre09.g416150.t1.2.html]

### Cre09.g416150.t1.2

Trivial name: ASA7  
SeqCoverage [median]: 69.60  
Mass: 22.20 kDa  
  
 

| Sequence | MissedCleavage | Mass [Da] | wtIntensity | muIntensity | Score |
| --- | --- | --- | --- | --- | --- |
| AHPDDLR | 0 | 822.40 | 6691.66 | 5053.69 | 107.67 |
| AHPDDLRK | 1 | 950.49 | 474.27 | 1493.10 | 70.91 |
| AYGKDYYYR | 1 | 1197.55 | 38708.43 | 37287.76 | 126.31 |
| FTFGSAADGPTASLGSNVK | 0 | 1825.88 | 68762.58 | 113420.42 | 321.85 |
| FYSMVDEFHR | 0 | 1329.58 | 31293.33 | 44302.08 | 166.66 |
| GSGKGVDVSVSAGAGSAK | 1 | 1532.78 | 5941.48 | 14043.19 | 107.98 |
| GVDVSVSAGAGSAK | 0 | 1203.61 | 90402.05 | 127153.74 | 244.06 |
| HNTVHPALGPATADGAVVQFLLAHAK | 0 | 2634.40 | 0.00 | 39013.79 | 93.40 |
| ISTAHSAFMNYLLTLTHER | 0 | 2204.10 | 0.00 | 44360.32 | 77.36 |
| KFYSMVDEFHR | 1 | 1457.68 | 1415.00 | 4977.67 | 53.21 |
| KVAASSLVLQDVSR | 1 | 1471.84 | 17972.31 | 35548.82 | 170.74 |
| VAASSLVLQDVSR | 0 | 1343.74 | 171719.87 | 332670.22 | 308.21 |
| VSYAPSDLR | 0 | 1006.51 | 25525.62 | 30712.63 | 147.36 |
| VSYAPSDLRK | 1 | 1134.60 | 114694.82 | 178560.34 | 134.57 |
| YSVLATWPDFTK | 0 | 1426.71 | 13523.28 | 23329.08 | 208.63 |
|  |  |  | 587124.7 | 1031926.8 |  |
